# Author Correction: Enhancing genome editing in hPSCs through dual inhibition of DNA damage response and repair pathways

**DOI:** 10.1038/s41467-024-50651-z

**Published:** 2024-07-29

**Authors:** Ju-Chan Park, Yun-Jeong Kim, Gue-Ho Hwang, Chan Young Kang, Sangsu Bae, Hyuk-Jin Cha

**Affiliations:** 1 College of Pharmacy, Seoul National University, Seoul, Republic of Korea; 2 College of Pharmacy and Research Institute of Pharmaceutical Sciences, Seoul National University, Seoul, Republic of Korea; 3 Genomic Medicine Institute, Seoul National University College of Medicine, Seoul, Republic of Korea; 4 Department of Biomedical Sciences, Seoul National University College of Medicine, Seoul, Republic of Korea; 5 Cancer Research Institute, Seoul National University College of Medicine, Seoul, Republic of Korea

**Keywords:** Stem-cell biotechnology, Embryonic stem cells, Targeted gene repair, CRISPR-Cas9 genome editing

Correction to: *Nature Communications* 10.1038/s41467-024-48111-9, published online 11 May 2024

In the Acknowledgements section of this article, the grant number relating to the Ministry of Food and Drug Safety was incorrectly given as #1475014063 and should have been 22202MFDS127. The original article has been corrected.

