# Supplementary material for: Enhancing genome editing in hPSCs through dual inhibition of DNA damage response and repair pathways
Source: Nat Commun. 2024 May 11;15:4002. doi: 10.1038/s41467-024-48111-9 (PMC11088699; doi:10.1038/s41467-024-48111-9)
Supplement: Supplementary file 1 — Supplementary information [file 41467_2024_48111_MOESM1_ESM.pdf]

Enhancing Genome Editing in hPSCs through Dual Inhibition of DNA Damage Response and Repair Pathways

Ju-Chan Park, Yun-Jeong Kim, Gue-Ho Hwang, Chan Young Kang, Sangsu Bae and Hyuk-Jin Cha

This PDF file includes:

Supplementary Tables

Supplementary Note

Supplemental Figures

Supplementary Tables

Supplementary Table 1. sgRNA sequence information

Target		Spacer (without PAM)
sgRNA	CCR5-3	GGCAGCATAGTGAGCCCAGA
	CCR5-10	GGTGACAAGTGTGATCACTT
	HPRT1-E4	GGGGACATAAAAGTAATTGG
	HPRT1-E6	GTATAATCCAAAGATGGTCA
	HEK2	GAACACAAAGCATAGACTGC
	HEK4	GGCACTGCGGCTGGAGGTGG
	RNF2	GTCATCTTAGTCATTACCTG
Nicking	HEK3	GATTGACCCAGGCCAGGGC
	HEK4	GACACACACACAGGCCTGG
	RNF2	TCAACCATTAAGCAAAACAT

Supplementary Table 2. pegRNA information

pegRNA	Spacer sequence	3' extension	PBS (nt)	RTT (nt)
HEK3_2c_1TtoA	GGCCCAGACTGA GCACGTGA	TCTGCCATCTCGTGCTCAG	9	10
HEK3_2e_1CTTins	GGCCCAGACTGA GCACGTGA	TCTGCCATCAAAGCGTGCTCAG	9	13
RNF2_2f_1GTAins	GTCATCTTAGTC ATTACCTG	AACGAACACCTCAGTACGTAATGACT AAGATGA	16	17
HEK3_2g_del1-5	GGCCCAGACTGA GCACGTGA	TGGAGGAAGCAGGGCTTCCTTTCCTC TGCCGTGCTCAG	9	29
HEK3_2g_del1-15	GGCCCAGACTGA GCACGTGA	TGGAGGAAGCAGGGCTTCCCGTGCTC AG	9	19
HEK4_G to T	GCACTGCGGCTG GAGGTGG	TTAACCCCAACCTCCAGCCGC	11	10
ACTB_AttB_ins	GCTATTCTCGCA GCTCACCA	GACGAGCGCGGCGATATCATCATCC ATGGatgatcctgacgacggagaccgccgtcgtcgaca agccTGAGCTGCGAGAA	13	67

Supplementary Table 3. Off-target information

HEK2	Sequence	Chromosome	Position
Target	GAACACAAAGCATAGACTGCGGG	chr5	87944779
OT1	ccACACCcAAGCATAGACTtCtGG	chr5	127049760
OT2	GAAtACtAAGCATAGACTcCaGG	chr4	52670026
OT3	GgAatCAAAGCAcAGACTGCaGG	chr18	24780735

HEK3	Sequence	Chromosome	Position
Target	GGCCCAGACTGAGCACGTGATGG	chr9	107422338
OT1	caCCCAGACTGAGCACGTGcTGG	chr15	79457572
OT2	aGCtCAGACTGAGCAaGTGAgGG	chr1	46540030
OT3	cagCCAGACaGAGCACGTGgaGG	chr9	134174097
OT4	GGCgCAGACaGAGCACGTGAcGa	chr11	134712518

OT5	GaCaCAGACcGgGCACGTGAgGG	chr7	67503052

CCR5-3	Sequence	Chromosome	Position
Target	GGCAGCATAGTGAGCCCAGAAGG	chr3	46373152
OT1	GGCAGCATAGTGctGCCCAGAGGG	chr2	1878938
OT2	GGCA-CATAGTGAGCCaAGATGG	chr5	99855972
OT3	GGCAGCA-AGTGAGgCCAGAAGG	chr13	18997106
OT4	GGaAGCA-AGTGAGCCCAGAAGG	chr13	95195396

RNF2	Sequence	Chromosome	Position
Target	GTCATCTTAGTCATTACCTG	chr1	185087634
OT1	GgtATCTaAGTCATTACCTG	chr5	92701252
OT2	GTCATCcTAGTCATTtaCTG	chr17	17168754
OT3	GTaATaTTAGTCATTACCgG	chr6	142890924
OT4	GTCATCTgAGgCATTAaCTG	chr6	150919927
OT5	GTaATCTgAGTCATTtCCTG	chr10	129047186

Supplementary Table 4. PCR primer information

Target	Sense	Anti-Sense
18srRNA	GTA ACC CGT TGA ACC CCA TT	CCA TCC AAT CGG TAG TAG CG
GAPDH	AAG GGT CAT CAT CTC TGC CC	GTC ATG GCA TGG ACT GTG GT
SOX2	TTC ACA TGT CCC AGC ACT ACC AGA	TCA CAT GTG TGA GAG GGG CAG TGT GC
LIN28	CCT TCC ATG TGC AGC TTA CTC	CAC GGT GCG GGC ATC TC
POU5F1	GTG GAG GAA GCT GAC AAC AA	ATT CTC CAG GTT GCC TCT CA
NANOG	GCA GAA GGC CTC AGC ACC TA	AGG TTC CCA GTC GGG TTC A

Supplementary Note

Gating and analysis strategy of Flow Cytometry data

For the EGFP positive population assay, we analyzed live cell based on SSC-A and FSC-A gating. The relative EGFP + Cell ratio was calculated by dividing each percentile data by average of Mock group. The live cell was gated based on the SSC-A and FSC-A values. The data was collected until the count of live cell gating of each sample reached to at least 5000, normally 10000. After the live cell gating, EGFP expression was discovered by FITC-A channel, and positive population was gated based on negative control(Normal H9), and positive control(EGFP constitutively expressing H9).

Supplemental Figures

Figure. S1

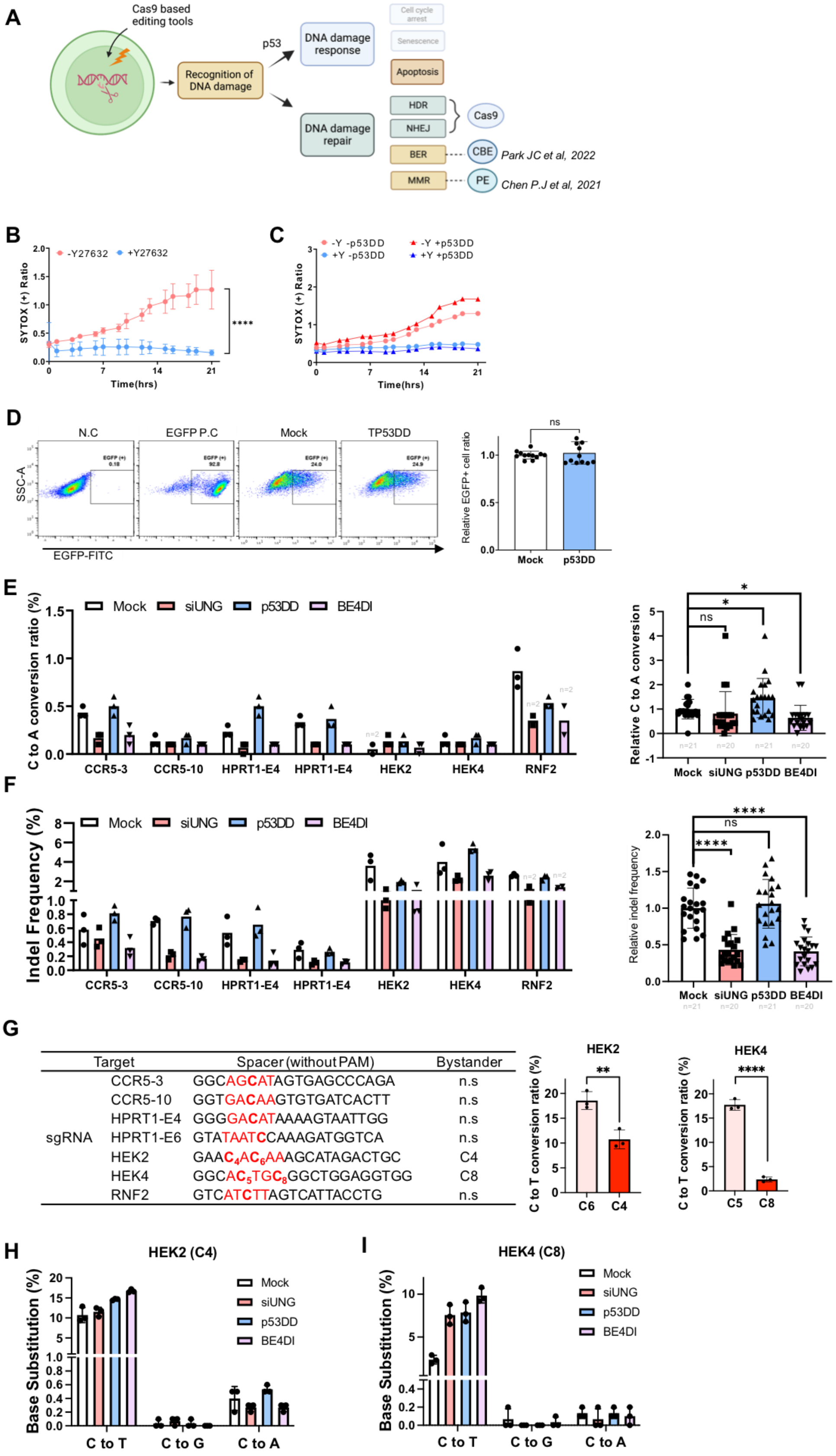

Target		Spacer (without PAM)	Bystander
sgRNA	CCR5-3	GGCAGCATAGTGAGCCCAGA	n.s
	CCR5-10	GGTGACAAGTGTGATCACTT	n.s
	HPRT1-E4	GGGGACATAAAAGTAATTGG	n.s
	HPRT1-E6	GTATAATCCAAAGATGGTCA	n.s
	HEK2	GAAC_4AC_6AAAGCATAGACTGC	C4
	HEK4	GGCAC_5TGC_8GGCTGGAGGTGG	C8
	RNF2	GTCATCTTAGTCATTACCTG	n.s

Figure S1 Dual inhibition of UNG and p53 improve CBE efficiency and product purity (A) Scheme of DNA damage response and DNA repair pathways followed by Cas9 based editing tools. Created with BioRender.com **(B-C)** Cell death after gene editing were tested by live cell imaging and SYTOX staining. The imaging started 1 hour after the electroporation (EP) to stabilize the cell. Effect of ROCK inhibitor Y27632 **(B)** and TP53DD **(C)** at EP induced cell death (n=2 for -Y+p53D and -Y+p53DD, n=3 for every other sample). **(D)** Transient expression of EGFP was analyzed by flow cytometry after 16 hrs of transfection with or without TP53DD vector. EGFP expressing hESCs after Mock or P53DD transfection were highlighted in flow cytometry, compared to negative (N.C, untransfected) and positive control (P.C, EGFP stably expressing hESCs). The EGFP positive cell population was represented in the right panel. (**E and F)** C to A substitution **(E)** and indel frequency **(F)** of BE4max with siRNA and pcDNA 3.0 vector (Mock), siRNA targeting UNG (siUNG), p53DD expression vector, and both siUNG and p53DD (BE4DI) at the indicated target sites. C to T and C to G conversion rates are presented in main figure 1B, D (n=3 except the designated replicates, n.d. for not detected data). **(G)** Sequence information of each target, Bases in the editing window are colored in red and target Cs are bolded (left). Multiple cytosines in the editing windows were numbered (left), The C to T editing efficiency of primary and second sites with BE4 was shown in bar graph (right) **(H and I)** C to T, C to G and C to A substitution ratio of C4 in HEK2 **(H)** and C8 in HEK4 **(I)** target. (n=3). n always represents the biologically independent samples if not else described. Bars represent mean values, and error bars represent the S.D. of independent biological replicates. Detailed information of statistical analysis are listed in the "Statistical analysis" section. The source data of B-I are provided in Source Data file.

Figure. S2

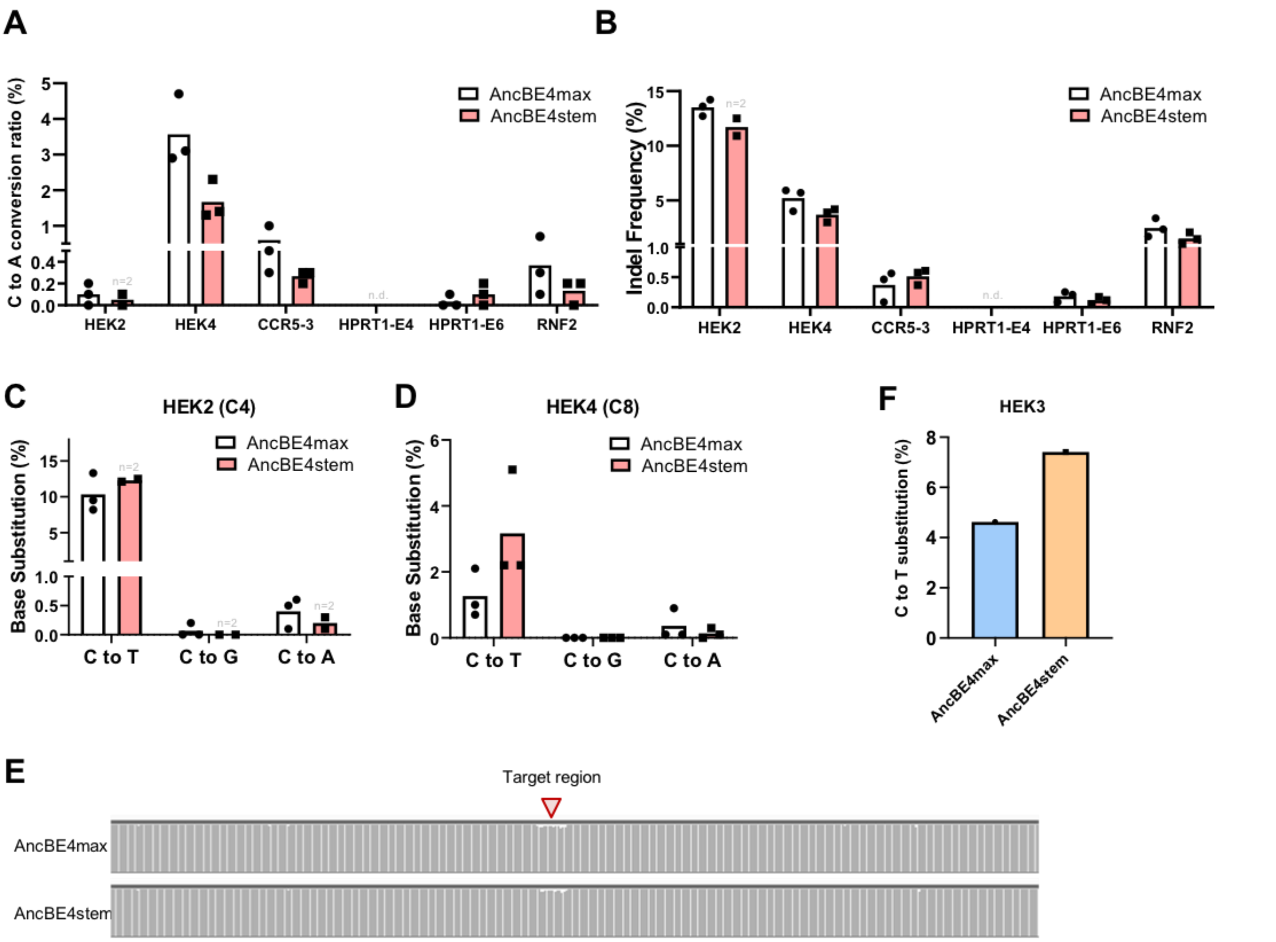

Figure S2 A single vector system for CBE dual inhibition strategy improves C to T conversion

(A-B) C to A substitution **(A)** and indel frequency **(B)** of AncBE4max and AncBE4stem at the indicated target sites. C to T and C to G conversion rates are presented in main figure 1B, D (n=3 except the designated replicates, n.d. for not detected data). **(C-D)** C to T, C to G and C to A substitution ratio of C4 in HEK2 **(C)** and C8 in HEK4 **(D)** target by AncBE4max or AncBE4stem (n=3 except the designated replicates). **(E)** IGV image of the RNF2 target treated with AncBE4max and AncBEstem analyzed by nanopore sequencing, shown in figure 2F. **(F)** HEK3 C to T substitution ratio by AncBE4max and AncBE4stem (n=1). n always represents the biologically independent samples if not else described. Bars represent mean values, and error bars represent the S.D. of independent biological replicates. Detailed information of statistical analysis are listed in the “Statistical analysis” section. The source data of A-D and F are provided in Source Data file.

Figure. S3

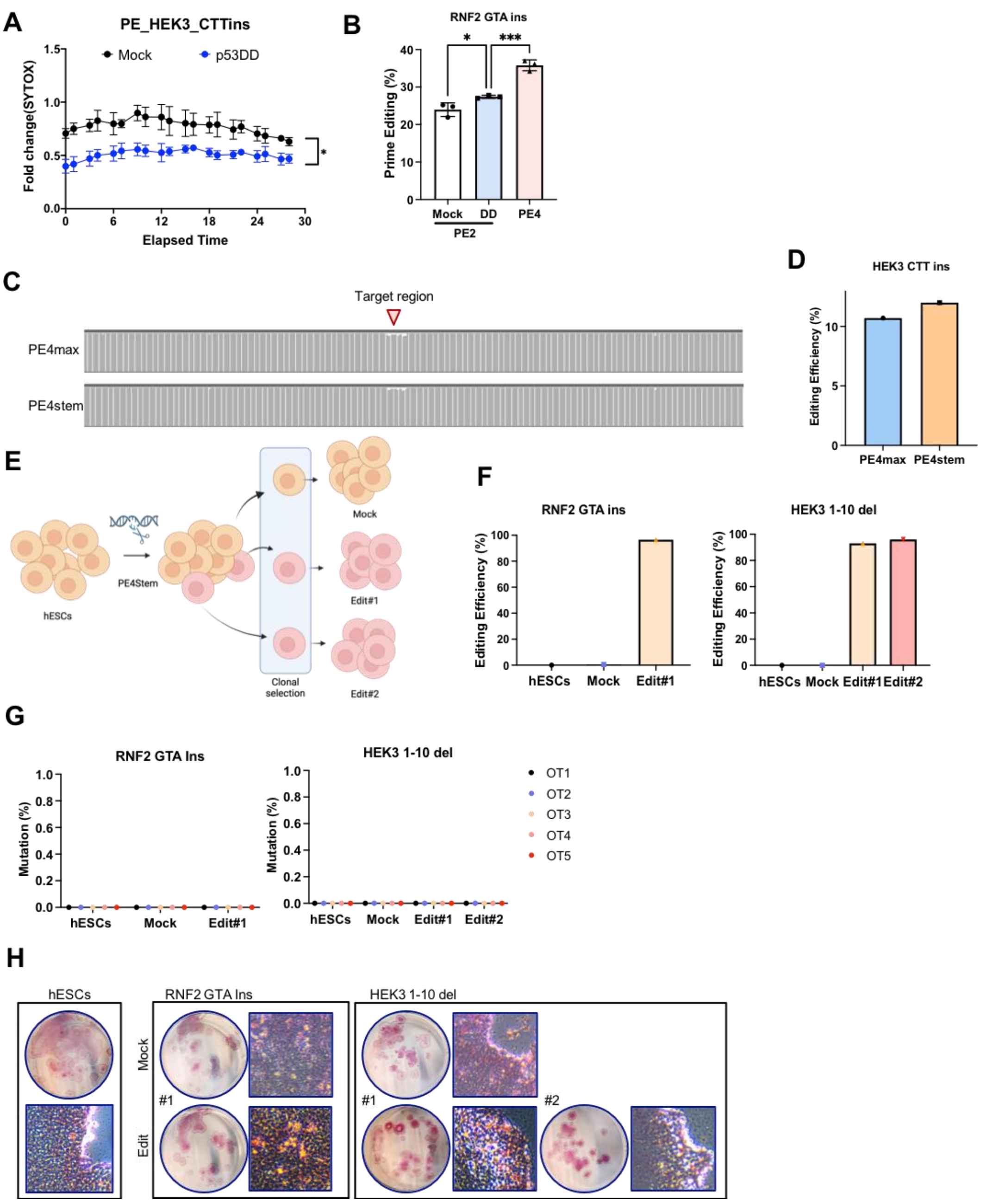

Figure S3 Dual inhibition of MMR and p53 pathway improves prime editing (A) Cell death after gene editing were tested by live cell imaging and SYTOX staining. The imaging was started after 24hrs of transfection of PEGRNA for CTT insertion of HEK3 without Y27632 treatment (n=3). **(B)** Prime editing efficiency of GTA insertion in RNF2 by PE2(Mock), PE2 with TP53DD (DD), and PE4. **(C)** IGV image of the RNF2 target treated with AncBE4max and AncBEstem analyzed by nanopore sequencing, targeted sample presented in figure 3J. **(D)** HEK3 CTT insertion efficiency of PE4max and PE4stem (n=1). **(E)** The graphical overview of establishment of single clone after

application of PE4stem. Untransfected control: hESCs, unedited control: Mock, and edited clones (Edit#1 and Edit#2). Created with BioRender.com **(F)** Editing efficiency of each clone for RNF2 GTA insertion (RNF2 GTA ins) and HEK3 1-10 deletion (HEK3 1-10del) (hESCs, Mock and Edit#1). (n=1). **(G)** Off-target editing of RNF2 and HEK3 target sites (OT1, OT2, OT3, OT4 and OT5) at each indicated clone (n=1). **(H)** Alkaline phosphatase staining in each indicated clone. (n=3). n always represents the biologically independent samples if not else described. Bars represent mean values, and error bars represent the S.D. of independent biological replicates. Detailed information of statistical analysis are listed in the "Statistical analysis" section. The source data of A-B, D, and F-H are provided in Source Data file.

Figure. S4

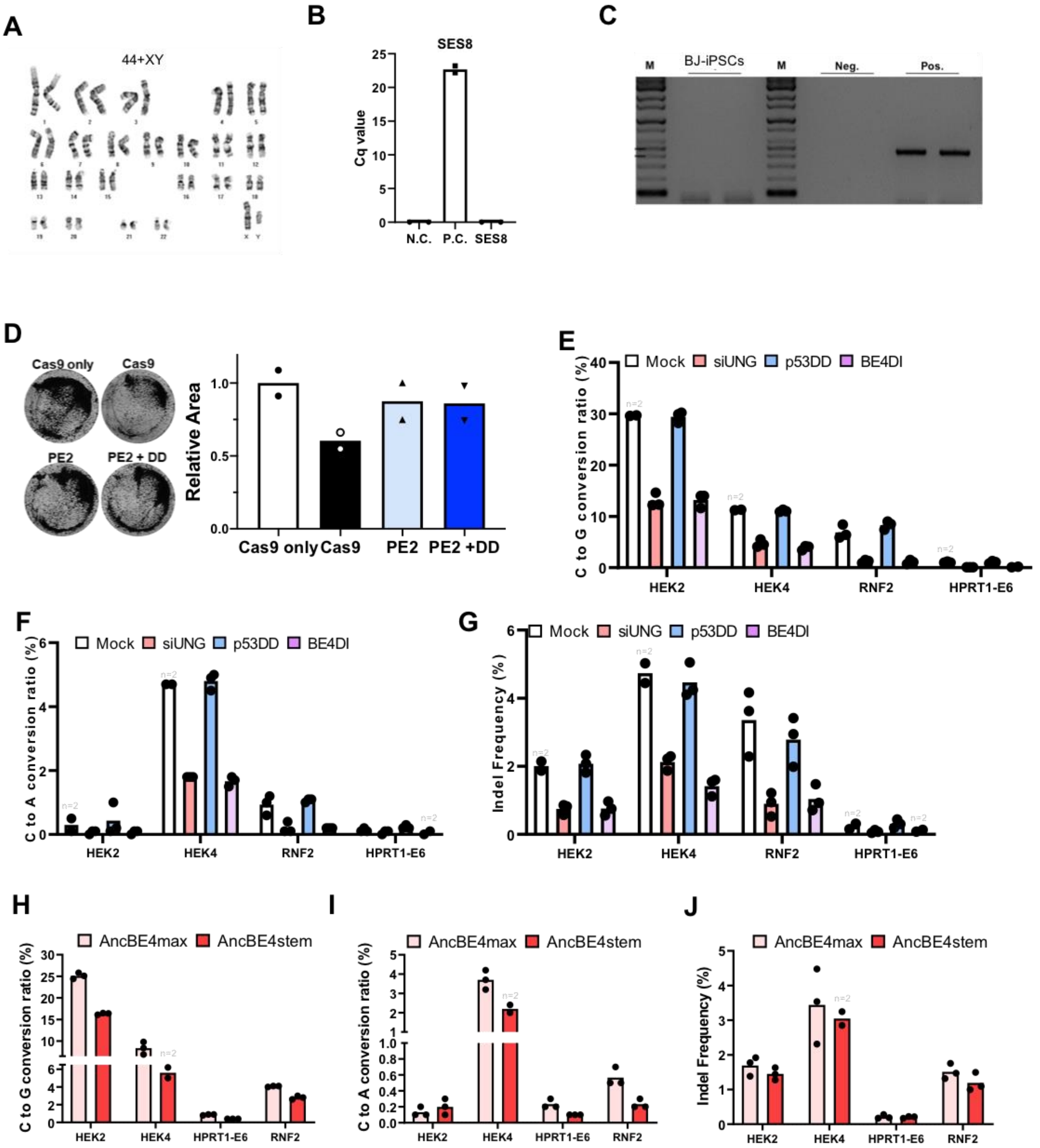

Figure S4 Diminished effect of p53DD on CBE and PE in p53 mutant hPSCs (A) G-Band karyotyping result of BJ-iPSCs (44+XY) **(B-C)** The mycoplasma was undetectable by mycoplasma specific primer PCR both in SES8 **(B)** and BJ-iPSCs (n=2) **(C). (D)** Colony area calculated after Cas9 and PE gene editing in TP53 Mut iPSCs (BJ-iPSCs). Colony area calculated by ImageJ after gene editing in normal cell (n=2)**. (E-G)** C to G substitution **(E)**, C to A substitution **(F)** ratio and indel

frequency **(G)** of AncBE4max siRNA and pcDNA 3.0 vector (Mock), siRNA targeting UNG (siUNG), p53DD expression vector, and both siUNG and p53DD (BE4DI) at the indicated target site on TP53DD mutant iPSCs (SES8) (n=3 except the designated replicates). **(H-J)** C to G substitution **(H)**, C to A substitution **(I)** ratio and indel frequency **(J)** of AncBE4max and AncBE4Stem at indicated endogenous targets in TP53DD mutant iPSCs (SES8). (n=3 except the designated replicates). n always represents the biologically independent samples if not else described. Bars represent mean values, and error bars represent the S.D. of independent biological replicates. Detailed informations of statistical analysis are listed in the "Statistical analysis" section. The source data of B and D-J are provided in Source Data file.

Figure. S5

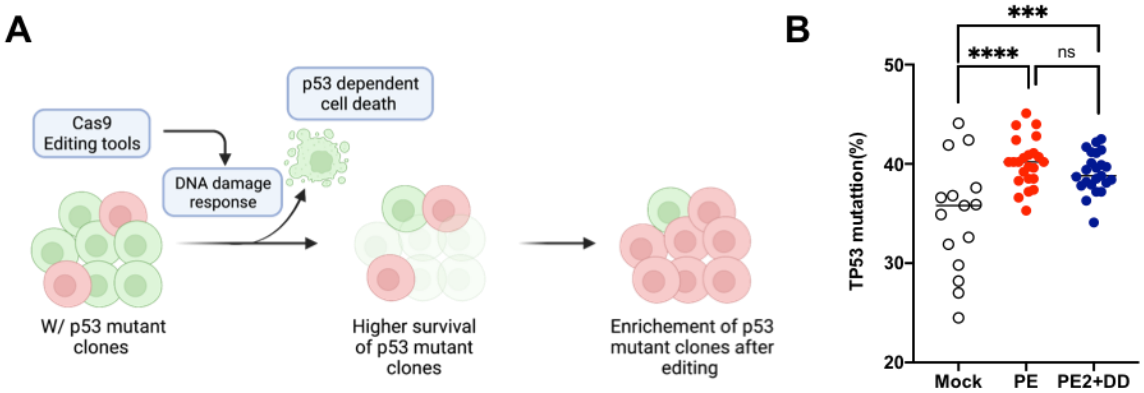

Figure S5 Enrichment of p53 mutant populations by prime editing (A) Graphical summary of DNA damage mediated enrichment of hPSCs harboring mutations in *TP53* gene, Normal cells are colored in green, hPSCs with p53 mutation are colored in red. Created with BioRender.com **(B)** *TP53* gene mutation R175H ratio analyzed by NGS after PE2 (PE) or PE2 with p53DD (PE2+DD) and pegRNA for HEK3. n always represents the biologically independent samples if not else described. Bars represent mean values, and error bars represent the S.D. of independent biological replicates. Detailed informations of statistical analysis are listed in the "Statistical analysis" section. The source data of B is provided in Source Data file.
