# Supplementary material for: Enhancing genome editing in hPSCs through dual inhibition of DNA damage response and repair pathways
Source: Nat Commun. 2024 May 11;15:4002. doi: 10.1038/s41467-024-48111-9 (PMC11088699; doi:10.1038/s41467-024-48111-9)
Supplement: Supplementary file 2 — Reporting Summary [file 41467_2024_48111_MOESM2_ESM.pdf]

nature portfolio

Corresponding author(s): Hyuk-Jin Cha

Last updated by author(s): Apr 14, 2024

Reporting Summary

Nature Portfolio wishes to improve the reproducibility of the work that we publish. This form provides structure for consistency and transparency in reporting. For further information on Nature Portfolio policies, see our Editorial Policies and the Editorial Policy Checklist.

Statistics

For all statistical analyses, confirm that the following items are present in the figure legend, table legend, main text, or Methods section.

n/a	Confirmed	
☐	☒	The exact sample size (*n*) for each experimental group/condition, given as a discrete number and unit of measurement
☐	☒	A statement on whether measurements were taken from distinct samples or whether the same sample was measured repeatedly
☐	☒	The statistical test(s) used AND whether they are one- or two-sided<br>*Only common tests should be described solely by name; describe more complex techniques in the Methods section.*
☐	☒	A description of all covariates tested
☐	☒	A description of any assumptions or corrections, such as tests of normality and adjustment for multiple comparisons
☐	☒	A full description of the statistical parameters including central tendency (e.g. means) or other basic estimates (e.g. regression coefficient) AND variation (e.g. standard deviation) or associated estimates of uncertainty (e.g. confidence intervals)
☐	☒	For null hypothesis testing, the test statistic (e.g. *F*, *t*, *r*) with confidence intervals, effect sizes, degrees of freedom and *P* value noted<br>*Give P values as exact values whenever suitable.*
☒	☐	For Bayesian analysis, information on the choice of priors and Markov chain Monte Carlo settings
☒	☐	For hierarchical and complex designs, identification of the appropriate level for tests and full reporting of outcomes
☒	☐	Estimates of effect sizes (e.g. Cohen's *d*, Pearson's *r*), indicating how they were calculated

Our web collection on statistics for biologists contains articles on many of the points above.

Software and code

Policy information about availability of computer code

Data collection	MinKNOW 23.07.11 was used to collect Nanopore sequencing data.<br>Miniseq Software Suite v2 was used to collect targeted deep sequencing data.
Data analysis	Off-target editing analysis of prime editing was conducted with Cas-Analyzer(http://www.rgenome.net/cas-analyzer/#!).<br>On-target and off-target editing analysis of base editing was conducted with BE-Analyzer((http://www.rgenome.net/be-analyzer/#!).<br>On-target editing analysis of prime editing was conducted with PE-Analyzer(http://www.rgenome.net/pe-analyzer/#!).<br>Statistical analysis was performed by GraphPad Prism(10.2.2).<br>The analysis pipeline for analysis of substitution is available at https://github.com/iamleohwang/BE_PE_Sub_analyze.

For manuscripts utilizing custom algorithms or software that are central to the research but not yet described in published literature, software must be made available to editors and reviewers. We strongly encourage code deposition in a community repository (e.g. GitHub). See the Nature Portfolio guidelines for submitting code & software for further information.

Data

Policy information about availability of data

All manuscripts must include a data availability statement. This statement should provide the following information, where applicable:

- Accession codes, unique identifiers, or web links for publicly available datasets
- A description of any restrictions on data availability
- For clinical datasets or third party data, please ensure that the statement adheres to our policy

High-throughput sequencing data have been deposited in the NCBI Sequence Read Archive database (https://www.ncbi.nlm.nih.gov/sra) BioProject #PRJNA1042664. RNA-seq data have been deposited in the Gene Expression Omnibus under accession GSE247589. Plasmids encoding AncBE4stem (Addgene no. 208766) and PE4stem (Addgene no. 208768) engineered in this work are available on Addgene at https://www.addgene.org. The analysis pipeline for analysis of substitution is available at https://github.com/iamleohwang/BE_PE_Sub_analyze.

Research involving human participants, their data, or biological material

Policy information about studies with human participants or human data. See also policy information about sex, gender (identity/presentation), and sexual orientation and race, ethnicity and racism.

Reporting on sex and gender	N/A
Reporting on race, ethnicity, or other socially relevant groupings	N/A
Population characteristics	N/A
Recruitment	N/A
Ethics oversight	N/A

Note that full information on the approval of the study protocol must also be provided in the manuscript.

Field-specific reporting

Please select the one below that is the best fit for your research. If you are not sure, read the appropriate sections before making your selection.

☒ Life sciences ☐ Behavioural & social sciences ☐ Ecological, evolutionary & environmental sciences

For a reference copy of the document with all sections, see nature.com/documents/nr-reporting-summary-flat.pdf

Life sciences study design

All studies must disclose on these points even when the disclosure is negative.

Sample size	Size of samples were not determined using statistical methods but were based on previously published studies. Size of samples for statistical analysis were documented in the manuscript.
Data exclusions	No data was excluded.
Replication	All experiments conducted in this study were replicated multiple times using the identical experimental protocol and each replication yielded consistent and successful results. All replicates are performed independently(biological repeat).
Randomization	As our study is to validate the effect of the dual inhibition of DNA damage response and DNA repair pathways in base and prime editing, randomization was not needed.
Blinding	As our study is to validate the effect of the dual inhibition of DNA damage response and DNA repair pathways in base and prime editing, there are no analysis requires blinding.

Reporting for specific materials, systems and methods

We require information from authors about some types of materials, experimental systems and methods used in many studies. Here, indicate whether each material, system or method listed is relevant to your study. If you are not sure if a list item applies to your research, read the appropriate section before selecting a response.

Materials & experimental systems

n/a	Involved in the study
☒	☐ Antibodies
☐	☒ Eukaryotic cell lines
☒	☐ Palaeontology and archaeology
☒	☐ Animals and other organisms
☒	☐ Clinical data
☒	☐ Dual use research of concern
☒	☐ Plants

Methods

n/a	Involved in the study
☒	☐ ChIP-seq
☐	☒ Flow cytometry
☒	☐ MRI-based neuroimaging

Eukaryotic cell lines

Policy information about cell lines and Sex and Gender in Research

Cell line source(s)	H9, WA09, WiCell Research institute, XX<br>SES8 iPSCs, established by lentiviral reprogramming(Lee TH, et al. Functional recapitulation of smooth muscle cells via induced pluripotent stem cells from human aortic smooth muscle cells. Circ Res 106, 120-128 (2010)), XY<br>BJ-iPSCs, established by episomal reprogramming (Bang JS, et al. Optimization of episomal reprogramming for generation of human induced pluripotent stem cells from fibroblasts. Anim Cells Syst (Seoul) 22, 132-139 (2018)), XY
Authentication	Work for pluripotent stem cell was reviewed and approved by the institutional Review Board at Seoul National University (SNU IRB protocol #2305/003-014).
Mycoplasma contamination	Cell lines used in this manuscript were free from mycoplasma contamination.
Commonly misidentified lines<br>(See ICLAC register)	There are no commonly misidentified lines used in this study.

Plants

Seed stocks	N/A
Novel plant genotypes	N/A
Authentication	N/A

Flow Cytometry

Plots

Confirm that:

☒ The axis labels state the marker and fluorochrome used (e.g. CD4-FITC).

☒ The axis scales are clearly visible. Include numbers along axes only for bottom left plot of group (a 'group' is an analysis of identical markers).

☒ All plots are contour plots with outliers or pseudocolor plots.

☒ A numerical value for number of cells or percentage (with statistics) is provided.

Methodology

Sample preparation	Cells were detached with Accutase and washed with DPBS
Instrument	BD FACS Calibur/Celesta Flow Cytometer
Software	FlowJo
Cell population abundance	Data was acquired up to 5000 cells in FSC/SSC positive population

Gating strategy	FSC/SSC gating was conducted to exclude debris. Negative control and EGFP positive control was used to determine GFP+ population gating.

☒ Tick this box to confirm that a figure exemplifying the gating strategy is provided in the Supplementary Information.
